# Methotrexate vs. Surgery in the Management of Ectopic Pregnancy: A Comparative Analysis of Treatment Outcomes

**DOI:** 10.7759/cureus.85169

**Published:** 2025-06-01

**Authors:** Asia Habib, Shumaila Sardar, Amina Iftikhar, Laiba LNU, Mahnoor Bokhari, Mah Rukh, Yusra LNU, Summaya Asmat, Bibi Asma, Muhammad Sikandar

**Affiliations:** 1 Department of Obstetrics and Gynecology, Rehman Medical Institute, Peshawar, PAK; 2 Department of Obstetrics and Gynecology Unit B, Medical Teaching Institute Hayatabad Medical Complex, Peshawar, PAK; 3 Department of Medicine, Ayub Teaching Hospital, Abbottabad, PAK; 4 Department of General Medicine, Rural Health Center Regi, Peshawar, PAK; 5 Department of Biochemistry, Khyber Girls Medical College, Peshawar, PAK

**Keywords:** adhesions, ectopic pregnancy, methotrexate, reproductive outcomes, surgical intervention, treatment success

## Abstract

Background: Methotrexate (MTX) is a non-invasive alternative to surgery for ectopic pregnancy, a condition in which a fertilized egg implants outside the uterine cavity and poses serious hazards to the mother.

Objective: This study aimed to compare the effectiveness, safety, and reproductive outcomes of MTX versus surgical intervention in the management of ectopic pregnancy.

Methodology: A two-year comparative study was conducted from 2022 to 2024, with 260 women diagnosed with unruptured ectopic pregnancies. Participants were randomized to either the surgical group (n = 130) or the MTX group (n = 130). The surgical group had either open or laparoscopic surgery, and MTX was given as a single dose. In addition to gathering laboratory, clinical, and demographic data, patients were monitored for three months. IBM SPSS Statistics for Windows, Version 26 (Released 2019; IBM Corp., Armonk, New York, United States) was used for statistical analysis.

Results: One hundred twenty-eight out of 130 patients (98.46%) in the surgical group had a successful course of therapy, while 114 out of 130 patients (87.69%) in the MTX group saw a favorable result (p=0.001). With a mean hospital stay of 1.2 ± 0.5 days, the MTX group had fewer problems than the surgical group, which saw a mean hospital stay of 3.0 ± 1.2 days (p<0.001). In terms of reproductive outcomes, 95 out of 130 patients (73.08%) in the surgical group and 89 out of 130 patients (68.46%) in the MTX group went on to get pregnant intrauterine (p=0.56).

Conclusion: Surgery has a greater success rate than MTX when it comes to treating ectopic pregnancy, but MTX has benefits in terms of hospital stay and recuperation after surgery. Treatment decisions need to be tailored to each patient's unique clinical situation.

## Introduction

Ectopic pregnancy, characterized by implantation of a fertilized ovum outside the uterine cavity, most commonly in the fallopian tube, remains a leading cause of maternal morbidity and first-trimester pregnancy-related mortality, affecting 1-2% of all pregnancies globally [1,2]. Historically, surgical management through salpingectomy or salpingostomy has been the cornerstone of treatment [3]. Salpingectomy involves removal of the affected fallopian tube, while salpingostomy preserves it by removing only the ectopic tissue.

However, the advent of methotrexate (MTX) therapy, a folate antagonist that inhibits DNA synthesis and trophoblastic proliferation, has dramatically transformed the treatment landscape [4]. This shift reflects a broader trend in gynecology toward minimally invasive, fertility-preserving, and patient-centered care [5,6]. MTX offers a non-surgical option that can preserve reproductive potential, avoid anesthesia, and reduce operative complications, especially when used in hemodynamically stable, early-stage unruptured ectopic pregnancies with low hCG levels (<5000 IU/L), no fetal cardiac activity, and no contraindications such as hepatic/renal dysfunction, immunodeficiency, or peptic ulcer disease [7,8].

Despite its advantages, MTX is not suitable for all patients. When contraindicated or if clinical instability arises, surgical intervention, particularly laparoscopic, is preferred and remains life-saving [9]. Surgical treatment also poses risks such as adhesions, tubal damage, and potential fertility impact [10,11]. A recent study by Yao et al. [12] stated that MTX should be considered as a viable alternative to surgical treatment. A meta-analysis by Long et al. [13] demonstrated that MTX is associated with a higher rate of future intrauterine pregnancies compared to surgery, without increasing the risk of recurrent ectopic pregnancy. Furthermore, MTX has shown comparable long-term fertility outcomes to conservative surgery, especially in women with previous infertility or reduced tubal reserve [14]. Emerging evidence suggests that MTX, when used appropriately, is associated with comparable long-term fertility outcomes to conservative surgery, especially in women with a prior history of infertility or limited tubal reserve [15].

Recent studies also support the expanding role of MTX in rare or atypical ectopic sites, such as rectal ectopic pregnancy (REP). A 2023 case report from Vietnam documented successful treatment of REP through ultrasound-guided local MTX injection and systemic multidose MTX, highlighting the utility of this conservative strategy in extremely rare and life-threatening presentations [16]. Similarly, retroperitoneal ectopic pregnancy, another rare form, was managed successfully with expectant monitoring alone, where spontaneous resolution occurred without surgical or medical intervention. This reinforces that MRI and precise imaging are critical for diagnosis and that non-interventional strategies may be appropriate for select nonviable and unruptured cases [17].

A 2023 individual participant data meta-analysis by Solangon et al. [18] compared MTX and expectant management in women with tubal ectopic pregnancies and low serum hCG levels (<2000 IU/L). The analysis demonstrated no statistically significant difference in treatment success rates, 79.3% for MTX versus 68.6% for expectant management, although MTX was associated with more adverse side effects. These findings support expectant management as a viable first-line approach in asymptomatic, hemodynamically stable patients with low and declining hCG levels, particularly when close monitoring is feasible. As the choice between medical, surgical, and expectant treatment remains multifactorial, taking into account patient preferences, clinical presentation, imaging findings, hCG levels, and available healthcare resources, it is critical to assess these strategies comparatively to inform individualized care. This study aims to compare the success rates, safety profiles, and reproductive outcomes of MTX versus surgical intervention in the management of ectopic pregnancy across a well-defined patient cohort.

## Materials and methods

Study design and setting

This prospective comparative study was conducted at Hayatabad Medical Complex, Peshawar, over a period of two years, from September 2022 to August 2024 to evaluate and compare the effectiveness, safety, and reproductive outcomes of MTX therapy versus surgical management in a well-defined cohort of women diagnosed with tubal ectopic pregnancy. Treatment success was defined as the complete resolution of ectopic pregnancy without the need for additional intervention. Safety outcomes included rates of complications such as tubal rupture, infection, and the need for transfusion. Reproductive outcomes assessed included future intrauterine pregnancy, time to conception, and recurrence of ectopic pregnancy. The study focused on hemodynamically stable women with confirmed tubal ectopic pregnancy, adnexal masses ≤3.5 cm, and baseline serum hCG levels ≤5000 IU/L, criteria commonly used to determine eligibility for MTX therapy. Surgical interventions were categorized into salpingectomy and salpingostomy based on intraoperative findings and fertility considerations.

Inclusion and exclusion criteria

Women aged 18 years or older diagnosed with tubal ectopic pregnancy confirmed by transvaginal ultrasonography and serum human chorionic gonadotropin (hCG) levels were included if they were hemodynamically stable (systolic blood pressure ≥100 mmHg, heart rate ≤100 bpm, and no requirement for vasopressor support). Additional eligibility criteria included adnexal mass size ≤3.5 cm, although patients with masses slightly larger than 3.5 cm were considered based on clinical judgment and patient preference. Baseline serum hCG levels had to be ≤5000 mIU/mL. Patients were required to provide informed consent and comply with scheduled follow-up visits.

Exclusion criteria included hemodynamic instability (systolic blood pressure <100 mmHg, heart rate >100 bpm, or vasopressor use), ruptured ectopic pregnancy necessitating immediate surgery, contraindications to MTX such as abnormal renal function (creatinine >1.4 mg/dL), hepatic dysfunction (transaminases >2 times upper limit of normal), significant blood dyscrasias (platelets <100,000/mm³, hemoglobin <9 g/dL), active pulmonary disease, breastfeeding, peptic ulcer disease, or immunodeficiency. Patients who declined the assigned treatment or were lost to follow-up were excluded from the final analysis.

Sample size and sampling technique

The sample size was estimated based on previous studies to achieve 80% power with a 5% significance level for detecting differences in treatment success rates between groups [19]. The minimum required sample size was calculated to be 238 patients, and a total of 260 patients were included to enhance statistical power and account for potential loss to follow-up. A total of 260 eligible patients were randomized in a 1:1 ratio using a computer-generated randomization sequence with allocation concealment to either the MTX or surgical intervention groups. Although patients were randomized, those refusing assigned treatment were counseled and excluded to maintain study integrity.

MTX protocol

Patients in the MTX group received a single intramuscular dose of 50 mg/m² body surface area per established clinical guidelines [20]. Serum hCG levels were measured on days 4 and 7 post-treatment. A second dose was administered if the hCG decrease was less than 15%. Patients were advised to avoid alcohol and folic acid supplements during therapy and were monitored for adverse effects such as stomatitis, fatigue, and gastrointestinal symptoms. Compliance was assessed during follow-up visits.

Surgical intervention

Surgical management comprised either laparoscopic or open salpingectomy or salpingostomy. The surgical approach and procedure choice were determined intraoperatively based on tubal condition and fertility considerations. Detailed intraoperative findings, postoperative recovery, complications, and histopathological confirmation of ectopic pregnancy were recorded.

Data collection

Clinical presentation, test values, and baseline demographic information were documented. A thorough obstetric and gynecological history was acquired, which included information on previous tubal operations, ectopic pregnancies, and fertility therapies. To track the effectiveness of the therapy and spot any issues, repeated ultrasound evaluations were carried out. Depending on clinical reasons, either open or laparoscopic surgery was performed on the surgical group. Postoperative outcomes including infection, pain, hospital stay, and complications such as hemorrhage and bowel injury were recorded. Adhesions were assessed intraoperatively in surgical cases and during follow-up visits through clinical examination and imaging (e.g., transvaginal ultrasound) when indicated. When appropriate, surgical tissues were used to acquire histopathological confirmation of ectopic pregnancy.

Follow-up and outcome measures

Patients were followed for a minimum of three months post-treatment. Follow-up included serial serum hCG measurements, ultrasound evaluations, and clinical assessments. Treatment success was defined as complete resolution of the ectopic pregnancy without additional medical or surgical intervention. Safety outcomes included rates of complications such as tubal rupture, infection, blood transfusion, and MTX-related side effects. In cases of MTX treatment failure, indicated by rising or plateauing hCG levels or persistent ectopic mass, patients received a second dose of MTX or underwent surgical intervention, based on clinical judgment. Reproductive outcomes monitored included return of normal menstrual cycles, time to conception, rates of future intrauterine pregnancy, and recurrence of ectopic pregnancy during the follow-up period.

Statistical analysis

IBM SPSS Statistics for Windows, Version 26 (Released 2019; IBM Corp., Armonk, New York, United States) was used to analyze the data. The chi-square test was used to compare categorical data, while the independent t-test was used to evaluate continuous variables. Logistic regression analysis was performed to identify predictors of treatment success or failure, including baseline serum hCG levels, adnexal mass size, previous ectopic pregnancy, and treatment type. Reproductive outcomes, complications, and treatment success rates were assessed. p-values less than 0.05 were regarded as statistically significant.

**Figure 1:**
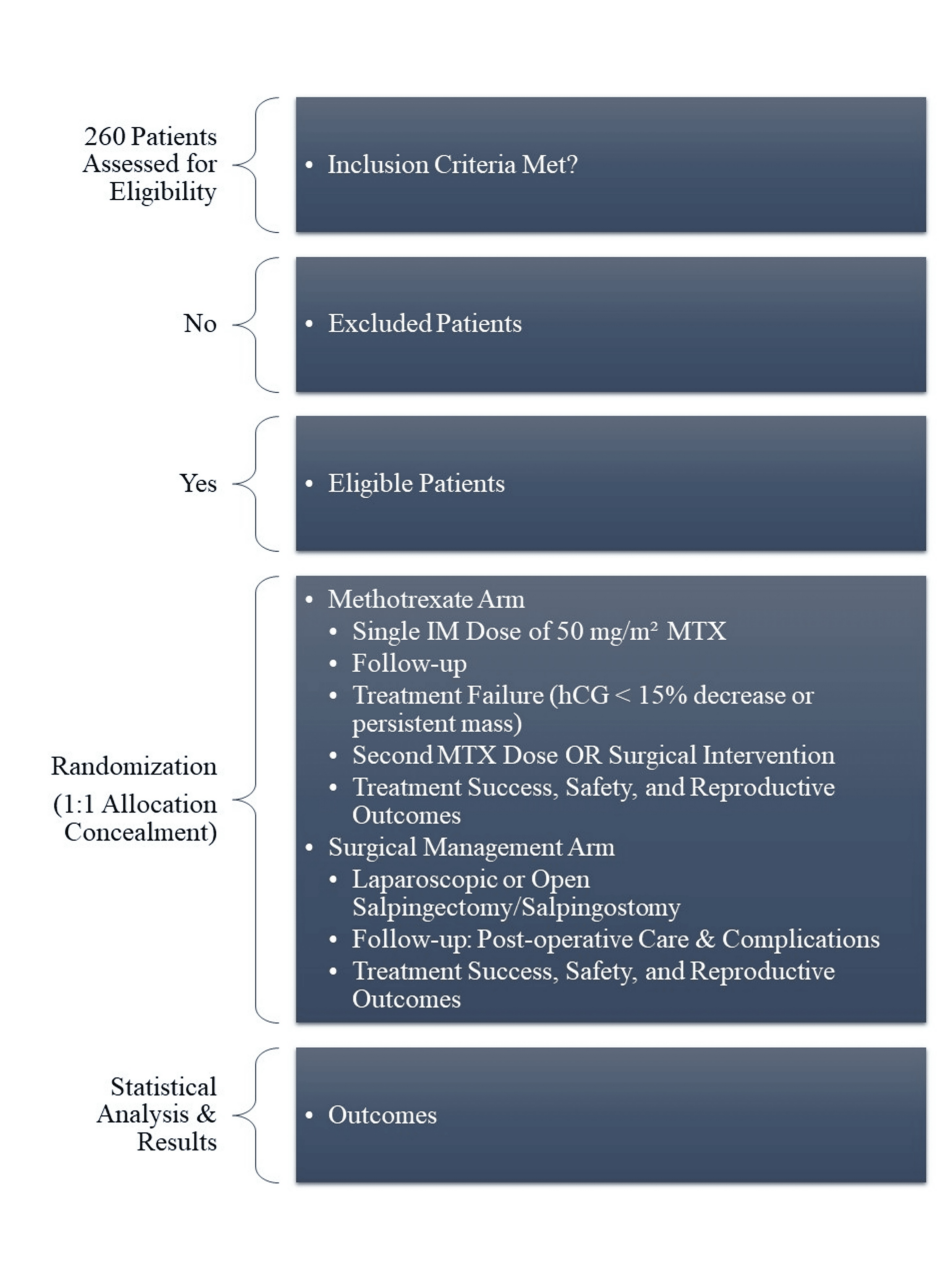
Flowchart Showing the Study Summary IM: Intramuscular; MTX: Methotrexate

A detailed flowchart illustrating the study design is provided in Figure 1. It outlines patient screening, application of inclusion and exclusion criteria, randomization into MTX or surgical treatment arms, and subsequent follow-up. The chart highlights key clinical decision points, such as eligibility for second MTX dosing or surgical intervention in cases of treatment failure. It also summarizes the evaluation of treatment success, safety, and reproductive outcomes, enhancing the replicability and transparency of our methodological approach.

## Results

A total of 260 participants were included in the study, with 130 in the MTX group and 130 in the surgical group (Table 1). The mean age was comparable between groups (28.5 ± 4.2 years for MTX vs. 29.2 ± 4.5 years for surgery). The majority of participants had one or two previous pregnancies (65 (50.00%) in the MTX group vs. 62 (47.69%) in the surgical group). The mean gestational age at diagnosis was similar (5.2 ± 1.1 weeks in the MTX group vs. 5.1 ± 1.0 weeks in the surgical group). The mean serum hCG levels were also comparable (3500 ± 1200 mIU/mL in the MTX group vs. 3600 ± 1100 mIU/mL in the surgical group). Most participants had an ectopic mass ≤3.5 cm (120 (92.31%) in the MTX group vs. 118 (90.77%) in the surgical group). Prior tubal surgery rates were 15 (11.54%) in the MTX group vs. 17 (13.08%) in the surgical group, while previous ectopic pregnancy rates were 30 (23.08%) vs. 28 (21.54%), respectively. The history of fertility treatment was also similar between groups (25 (19.23%) vs. 22 (16.92%)).

**Table 1:** Demographic, Clinical, and Laboratory Characteristics of Participants

Characteristic		Methotrexate Group (n=130)	Surgical Group (n=130)
Age in Years	(mean ± SD)	28.5 ± 4.2	29.2 ± 4.5
Parity (n, %)	0	50 (38.46%)	48 (36.92%)
	1-2	65 (50.00%)	62 (47.69%)
	≥3	15 (11.54%)	20 (15.38%)
Gestational Age (weeks)	(mean ± SD)	5.2 ± 1.1	5.1 ± 1.0
hCG Levels (mIU/mL)	(mean ± SD)	3500 ± 1200	3600 ± 1100
Size of Ectopic Mass (cm)	≤3.5 cm	120 (92.31%)	118 (90.77%)
	>3.5 cm	10 (7.69%)	12 (9.23%)
Previous Ectopic Pregnancy (n, %)	Yes	30 (23.08%)	28 (21.54%)
	No	100 (76.92%)	102 (78.46%)
Prior Tubal Surgery (n, %)	Yes	15 (11.54%)	17 (13.08%)
	No	115 (88.46%)	113 (86.92%)
Fertility Treatment History (n, %)	Yes	25 (19.23%)	22 (16.92%)
	No	105 (80.77%)	108 (83.08%)

**Figure 2:**
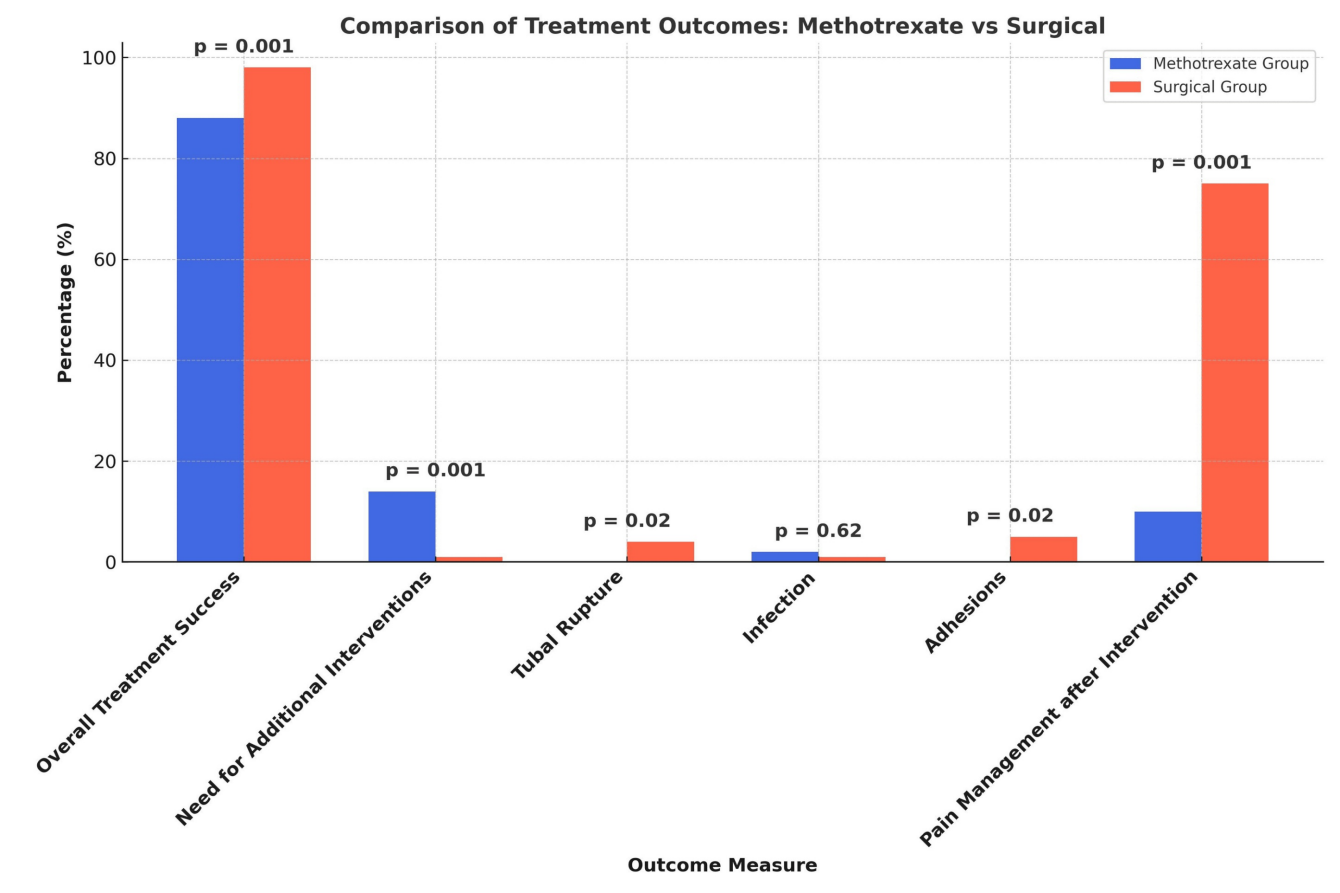
Treatment Success, Intraoperative Findings, and Postoperative Recovery *p-value <0.05 is significant.

The overall treatment success rate was significantly higher in the surgical group (128 (98.46%) vs. 114 (87.69%) in the MTX group, p = 0.001), and more patients in the MTX group required additional interventions (16 (12.31%) vs. 2 (1.54%), p = 0.001), as shown in Figure 2. None of the patients in the MTX group experienced tubal rupture, whereas five (3.85%) patients in the surgical group had a rupture (p = 0.02). Adhesions were more frequent following surgery (8 (6.15%), p = 0.02), while infection rates were comparable (2 (1.54%) in the MTX group vs. 3 (2.31%) in the surgical group, p = 0.62). The need for pain management after intervention was significantly lower in the MTX group (12 (9.23%) vs. 98 (75.38%) in the surgical group, p = 0.001), and the hospital stay was shorter (1.2 ± 0.5 days for MTX vs. 3.0 ± 1.2 days for surgery, p = 0.001).

At the three-month follow-up, the rates of subsequent intrauterine pregnancy were similar between the MTX (89 (68.46%)) and surgical groups (95 (73.08%), p = 0.56) (Table 2). The rate of recurrent ectopic pregnancy was low in both groups (5 (3.85%) in the MTX group vs. 4 (3.08%) in the surgical group, p = 0.67). Ovarian cysts were uncommon, with two (1.54%) cases in the MTX group and one (0.77%) in the surgical group (p = 0.69). However, adhesions were significantly more frequent in the surgical group (6 (4.62%), p = 0.02). The pregnancy loss rates (6 (4.62%) in the MTX group vs. 5 (3.85%) in the surgical group, p = 0.67) and the need for IVF treatment (12 (9.23%) in the MTX group vs. 10 (7.69%) in the surgical group, p = 0.65) were comparable between groups.

**Table 2:** Follow-Up and Reproductive Outcomes (Three-Month Follow-Up) *P-value <0.05 is significant.

Reproductive Outcome		Methotrexate Group (n=130)	Surgical Group (n=130)	p-value*
Subsequent Intrauterine Pregnancy	(n, %)	89 (68.46%)	95 (73.08%)	0.56
Subsequent Ectopic Pregnancy	(n, %)	5 (3.85%)	4 (3.08%)	0.67
Fertility-Related Complications	Ovarian Cysts	2 (1.54%)	1 (0.77%)	0.69
	Adhesions	0 (0.00%)	6 (4.62%)	0.02
Need for IVF Treatment	Yes	12 (9.23%)	10 (7.69%)	0.65
	No	118 (90.77%)	120 (92.31%)	0.65
Pregnancy Loss	(n, %)	6 (4.62%)	5 (3.85%)	0.67

Complications and adverse events varied between the groups, reflecting the nature of each intervention (Table 3). In the surgical group, major complications included hemorrhage requiring transfusion in four (3.08%) patients (p = 0.04) and one (0.77%) case of bowel injury. Adhesion formation was significantly more frequent following surgery, observed in six (4.62%) patients (p = 0.02). Infection rates were comparable between the MTX (2 (1.54%)) and surgical (3 (2.31%)) groups (p = 0.62). Conversely, adverse drug reactions specific to MTX were observed in 8 (6.15%) patients in the MTX group. These cases included nausea (3, 2.31%), stomatitis (2, 1.54%), transient liver enzyme elevation (2, 1.54%), and fatigue (10.77%). No drug-related adverse events were observed in the surgical group.

**Table 3:** Complications and Adverse Events *p-value <0.05 is significant. **Although adhesion formation was significantly more frequent in the surgical group, the three-month follow-up duration may not fully capture long-term fertility implications. Future studies with extended follow-up are warranted to assess these outcomes comprehensively.

Complication/Adverse Event		Methotrexate Group (n=130)	Surgical Group (n=130)	p-value*
Major Complications	Hemorrhage (requiring transfusion)	0 (0.00%)	4 (3.08%)	0.04
	Bowel Injury	0 (0.00%)	1 (0.77%)	0.32
Minor Complications	Infection	2 (1.54%)	3 (2.31%)	0.62
	Adhesions**	0 (0.00%)	6 (4.62%)	0.02
Adverse Drug Reactions (Methotrexate)	Nausea	3 (2.31%)	0 (0.00%)	-
	Stomatitis	2 (1.54%)		
	Transient elevation of liver enzymes	2 (1.54%)		
	Fatigue	1 (0.77%)		
	Total	8 (6.15%)		

The logistic regression analysis of treatment success predictors is summarized in Table 4. The odds ratio (OR) for age ≤30 years was 0.82 (95% CI: 0.51-1.32, p = 0.43), with 90 (69.23%) patients in the MTX group and 95 (73.08%) in the surgical group under 30 years old. The OR for hCG levels ≤3000 mIU/mL was 1.16 (95% CI: 0.70-1.93, p = 0.58), with 95 (73.08%) patients in the MTX group and 100 (76.92%) in the surgical group falling within this range. The size of the ectopic mass (≤3.5 cm) was a significant predictor of treatment success, with an OR of 2.20 (95% CI: 1.12-4.34, p = 0.02) (120 (92.31%) in the MTX group vs. 118 (90.77%) in the surgical group). Nulliparity was comparable between groups (50 (38.46%) vs. 48 (36.92%), OR: 1.05, 95% CI: 0.72-1.53, p = 0.78). Lastly, gestational age ≤6 weeks was not a significant predictor, with OR: 1.25 (95% CI: 0.72-2.15, p = 0.43) (110 (84.62%) in the MTX group vs. 115 (88.46%) in the surgical group).

**Table 4:** Statistical Analysis of Predictors of Treatment Success OR: Odds Ratio; CI: Confidence Interval; hCG: Human Chorionic Gonadotropin; cm: centimeters; mIU/mL: milli-international units per milliliter. Multivariate logistic regression was performed using variables with p < 0.10 in univariate analysis. A p-value < 0.05 was considered statistically significant.

Predictor		Methotrexate Group (n=130)	Surgical Group (n=130)	Univariate OR (95% CI)	p-value	Multivariate OR (95% CI)	p-value
Age	(≤30 years vs. >30 years)	90 (69.23%)	95 (73.08%)	0.82 (0.51–1.32)	0.43	0.89 (0.53–1.48)	0.65
hCG Levels	(≤3000 vs. >3000 mIU/mL)	95 (73.08%)	100 (76.92%)	1.16 (0.70–1.93)	0.58	1.24 (0.72–2.15)	0.43
Size of Ectopic Mass	(≤3.5 cm vs. >3.5 cm)	120 (92.31%)	118 (90.77%)	2.20 (1.12–4.34)	0.02	2.05 (1.01–4.16)	0.04
Parity	Nulliparous	50 (38.46%)	48 (36.92%)	1.05 (0.72–1.53)	0.78	1.11 (0.72–1.72)	0.64
	Parous (1–2)	65 (50.00%)	62 (47.69%)				
	Parous (≥3)	15 (11.54%)	20 (15.38%)				
Gestational Age	(≤6 weeks vs. >6 weeks)	110 (84.62%)	115 (88.46%)	1.25 (0.72–2.15)	0.43	1.18 (0.66–2.13)	0.57

Table 5 shows no significant differences between the MTX and surgical groups regarding prior tubal surgery (15 [11.54%] vs. 17 [13.08%], p = 0.85), previous ectopic pregnancy (30 [23.08%] vs. 28 [21.54%], p = 0.88), history of fertility treatment (25 [19.23%] vs. 22 [16.92%], p = 0.74), or infection-related complications (2 [1.54%] vs. 3 [2.31%], p = 0.62). Additionally, the rates of subsequent intrauterine pregnancy at three-month follow-up were comparable between groups (89 [68.46%] vs. 95 [73.08%], p = 0.49).

**Table 5:** Comparison of Categorical Variables Between Methotrexate and Surgical Groups Using the Chi-Square Test *p-value <0.05: is significant. χ²: chi-square.

Variable		Methotrexate Group (n=130)	Surgical Group (n=130)	χ²	p-value*
Previous Ectopic Pregnancy	Yes	30 (23.08%)	28 (21.54%)	0.02	0.88
	No	100 (76.92%)	102 (78.46%)		
Prior Tubal Surgery	Yes	15 (11.54%)	17 (13.08%)	0.04	0.85
	No	115 (88.46%)	113 (86.92%)		
Fertility Treatment History	Yes	25 (19.23%)	22 (16.92%)	0.10	0.74
	No	105 (80.77%)	108 (83.08%)		
Complications (infection)	Yes	2 (1.54%)	3 (2.31%)	0.00	0.62
	No	128 (98.46%)	127 (97.69%)		
Subsequent Intrauterine Pregnancy	Yes	89 (68.46%)	95 (73.08%)	0.46	0.49
	No	41 (31.54%)	35 (26.92%)		

Table 6 indicates that there were no significant differences between the MTX and surgical groups in terms of ectopic mass size (p=0.41), hCG levels (p=0.45), gestational age (p=0.62), or age (p=0.24), according to independent t-test analysis. Nonetheless, the surgical group's hospital stay was much longer (3.0 ± 1.2 vs. 1.2 ± 0.5 days, p < 0.001).

**Table 6:** Comparison of Continuous Variables Between Methotrexate and Surgical Groups Using the Independent t-Test *p-value <0.05 is significant.

Variable	Methotrexate Group (n=130)	Surgical Group (n=130)	t-value	p-value*
Age (mean ± SD)	28.5 ± 4.2	29.2 ± 4.5	1.17	0.24
Gestational Age (weeks)	5.2 ± 1.1	5.1 ± 1.0	0.5	0.62
hCG Levels (mIU/mL)	3500 ± 1200	3600 ± 1100	0.76	0.45
Size of Ectopic Mass (cm)	3.2 ± 0.6	3.3 ± 0.5	0.83	0.41
Hospital Stay (days)	1.2 ± 0.5	3.0 ± 1.2	14.25	<0.001

## Discussion

Our study comparing MTX and surgical treatment for ectopic pregnancy demonstrated significant differences in treatment outcomes and associated side effects. The surgical group exhibited a markedly higher overall treatment success rate (98.46%) compared to the MTX group (87.69%, p = 0.001). This aligns with prior research showing that surgical intervention typically results in more immediate resolution of ectopic pregnancies, especially in cases involving hemodynamic instability or larger ectopic masses [21]. Consistently, studies by Sowter et al. [22] and Mol et al. [23] reported laparoscopic surgery success rates around 93%, outperforming MTX’s 65%.

Over the past quarter-century, management of ectopic pregnancy has shifted from primarily surgical approaches to more conservative, medical therapies, particularly with MTX. Advances in early diagnosis via sensitive β-hCG assays and transvaginal ultrasound have allowed clinicians to identify candidates likely to benefit from medical management. MTX is associated with high success rates ranging from 71% to 100%, reduced morbidity, cost savings (approximately $3,000 per patient), and patient preference, particularly in centers equipped with structured protocols and advanced diagnostics [21].

A randomized controlled trial comparing single-dose systemic MTX (50 mg/m²) to laparoscopic surgery for unruptured tubal pregnancies found laparoscopic surgery to have superior success rates (93% vs. 65%), though MTX was better tolerated physically [22]. Failures of MTX were linked to higher β-hCG levels, with treatment recommended primarily for clinically stable women exhibiting mild symptoms and β-hCG concentrations below 5000 IU/L. A systematic review and meta-analysis of 15 trials further supported that laparoscopic salpingostomy, while slightly less effective than open surgery, is more cost-effective [23]. Systemic MTX, especially multi-dose regimens, achieved comparable success in selected patients with low β-hCG levels, and single-dose MTX was notably cost-effective when β-hCG was below 1500 IU/L. Fertility outcomes following treatment were similar across modalities, though expectant management lacked sufficient evidence for firm conclusions.

Despite the higher immediate success of surgery, the MTX group demonstrated significant benefits in terms of invasiveness and postoperative care. Fewer patients in the MTX group required additional postoperative treatments (1.54% vs. 12.31%, p = 0.001), underscoring the less invasive nature of medical therapy. Previous studies have highlighted MTX as a non-invasive option that may preserve fertility while avoiding anesthesia and surgical risks, especially in early, unruptured ectopic pregnancies [24]. Our logistic regression analysis reinforced the importance of ectopic mass size as a predictor of treatment success (OR: 2.05, p = 0.04), suggesting that patients with ectopic masses ≤3.5 cm are optimal candidates for MTX therapy.

Regarding complications, infection rates did not differ significantly between groups (1.54% vs. 2.31%, p = 0.62). However, the surgical group exhibited a higher risk of tubal rupture (3.85% vs. 0%, p = 0.02), consistent with other studies reporting increased intraoperative complications due to the invasive nature of surgery and fragile tissue in advanced ectopic pregnancies [25]. The absence of tubal rupture in the MTX group supports the notion that medical management reduces severe adverse effects, making it a safer option for carefully selected patients.

A notable finding was the significantly shorter hospital stay in the MTX group (1.2 ± 0.5 days vs. 3.0 ± 1.2 days, p < 0.001), highlighting the quicker recovery and non-invasive benefits of medical therapy. This is consistent with the literature demonstrating faster convalescence associated with MTX treatment [26]. Reduced hospitalization and lower postoperative pain medication requirements (9.23% vs. 75.38%, p = 0.001) further contribute to decreased healthcare costs and improved patient comfort [20,21].

In terms of reproductive outcomes, both groups showed comparable rates of subsequent intrauterine pregnancy (68.46% MTX vs. 73.08% surgery, p = 0.56), corroborating evidence that MTX does not adversely affect future fertility compared to surgical intervention [23]. Although surgical treatment carries risks of tubal damage, the similarity in pregnancy rates suggests both approaches preserve fertility when applied appropriately. Adhesions were more common after surgery (4.62% vs. 0%, p = 0.02), which aligns with other reports linking postoperative adhesions to potential infertility and recurrent ectopic pregnancies [27]. These findings underscore the importance of meticulous surgical technique and patient selection to minimize long-term reproductive complications.

While surgical intervention offers a higher immediate success rate, MTX presents a viable alternative with fewer side effects, faster recovery, and comparable long-term reproductive outcomes in properly selected patients. Therefore, individualized treatment planning, considering patient stability, ectopic size, and fertility goals, is crucial to optimizing clinical outcomes.

Strengths and limitations

The primary strength of this study is the randomized allocation of patients to surgical or MTX treatment groups, which minimized selection bias and ensured baseline comparability, thereby enhancing internal validity. Additionally, the relatively large sample size (n=260) improved the statistical power and precision of the findings. Conducted at a major tertiary care hospital serving a diverse patient population, the study benefits from consistent clinical protocols, increasing the reliability and generalizability of the results.

Nonetheless, certain limitations exist. The follow-up period of three months is sufficient to assess early outcomes and initiation of intrauterine pregnancy; however, it may not capture long-term fertility or recurrence risk. Furthermore, being a single-center study, regional or institutional factors could have influenced the results. Future multicenter studies with extended follow-up are warranted to confirm these findings and provide a more comprehensive understanding of the comparative effectiveness and safety of treatment modalities for ectopic pregnancy.

## Conclusions

This prospective comparative study highlights that both MTX therapy and surgical intervention are effective treatment modalities for tubal ectopic pregnancy in carefully selected, hemodynamically stable patients. Surgical management was associated with a significantly higher overall treatment success rate and reduced need for additional interventions. Conversely, MTX therapy offered notable advantages in terms of shorter hospital stays, lower incidence of adhesions, and reduced postoperative pain management requirements, reflecting its less invasive nature. These findings underscore the necessity of individualized treatment decisions, guided by clinical presentation, serum hCG levels, adnexal mass size, patient preferences, and fertility considerations.

Importantly, short-term reproductive outcomes at the three-month follow-up-including the incidence of subsequent intrauterine pregnancies and recurrent ectopic pregnancies-were comparable between the two groups. The limited duration of follow-up restricts definitive conclusions regarding long-term fertility outcomes and delayed complications, particularly adhesion-related infertility observed more frequently in the surgical group. In comparison with recent meta-analytic evidence supporting expectant management in low-risk cases, our findings emphasize the multifactorial nature of treatment selection. Future large-scale, longitudinal studies with extended follow-up are warranted to further elucidate the long-term reproductive implications and refine clinical guidelines for the optimal management of ectopic pregnancy.
